# Effects of Autologous Conditioned Serum, Autologous Protein Solution, and Triamcinolone on Inflammatory and Catabolic Gene Expression in Equine Cartilage and Synovial Explants Treated With IL-1β in Co-culture

**DOI:** 10.3389/fvets.2020.00323

**Published:** 2020-06-26

**Authors:** Ana Velloso Alvarez, Lindsey H. Boone, Satyanarayana R. Pondugula, Fred Caldwell, Anne A. Wooldridge

**Affiliations:** ^1^Department of Clinical Sciences, Auburn University, Auburn, AL, United States; ^2^Department of Anatomy, Physiology and Pharmacology, Auburn University, Auburn, AL, United States

**Keywords:** autologous conditioned serum, autologous protein solution, triamcinolone, osteoarthritis, equine, co-culture, joint disease

## Abstract

Autologous conditioned serum (ACS) and autologous protein solution (APS) are newer therapeutic options for osteoarthritis (OA). Co-culture of cartilage and synovium stimulated with IL-1β produces a similar physiologic response to tissues from naturally-ocurring OA. The study objective was to investigate the effects of ACS, APS, and triamcinolone (TA) on inflammatory and catabolic gene expression of inflamed joint tissues in co-culture. Blood was collected and processed for ACS and APS from six horses. Cartilage and synovial explants were harvested from the stifle, placed in co-culture, and treated as: (1) unstimulated control (2) stimulated control (3) ACS at 25% v/v (4) ACS at 50% v/v (5) APS at 25% v/v (6) APS at 50% v/v, (7) TA (10^−6^ M). Treatment groups 2–7 were stimulated with IL-1β (10 ng/ml). Cultures were maintained for 96 hours, and then both media and explants were harvested for measurement of gene expression and protein. IL-1β stimulation significantly increased IL-1β (*p* = 0.029), IL-8 (*p* = 0.011) and MMP-3 (*p* = 0.043) expression in synovium and IL-1β (*p* = 0.003) and TNF-α (*p* = 0.001) expression in cartilage. Treatment with 50% ACS and APS v/v downregulated IL-1β expression in cartilage more than TA treatment (*p* = 0.001 and *p* = 0.0004) and APS downregulated MMP-1 expression in synovial membrane (*p* = 0.025). Treatment with ACS and APS caused a trend in upregulation of IL-10 expression in synovium and type II collagen and aggrecan expression in cartilage. PGE_2_ media concentrations were significantly reduced following treatment with APS (13.7-fold decrease, *p* = 0.0001) and ACS (4.13-fold decrease, *p* = 0.024); while TA did not reduce PGE_2_ significantly (2.3-fold decreased *p* = 0.406). As disease-modifying therapies, ACS and APS modified the cellular response from synovial membrane and articular cartilage. ACS and APS may offer an improved strategy to improve clinical signs of horses with naturally occurring OA, compared to TA treatment.

## Introduction

Lameness due to osteoarthritis (OA) is a leading cause of reduced or lost performance in horses, placing a significant economic hardship on the equine industry (1, 2). OA not only affects equine athletes, it has been shown to affect more than 80% of the equine geriatric population (3). Currently, the mainstay of intra-articular OA therapy is modifying the symptoms of disease through temporary reduction of inflammation via administration of corticosteroids with or without viscosupplementation (4).

The most commonly used corticosteroid by equine practitioners in high-motion joints is triamcinolone acetonide (TA) (4). TA has been shown to be chondroprotective in *in vitro* studies (5–7), however, there is still concern about the effects of repeated, long term use of TA and other corticosteroids on cartilage. Therefore, intra-articular biologics may be preferred over corticosteroids when cost is not an issue or horses have become non-responsive to corticosteroid treatment (4). Several blood-derived orthobiologic products targeted at disease modification, such as autologous conditioned serum (ACS) and autologous protein solution (APS), are expanding the therapeutic options for clinicians treating horses with joint-related injury. Both products are obtained from the patient's blood and administered directly into the affected joint(s) for the treatment of OA. The cellular and protein profile of ACS and APS have been characterized independently in several studies (8–10). Currently, only one study has compared the anti-inflammatory cytokine and growth factor concentration in ACS and APS collected from the same horse, finding APS had higher concentrations of TGF-β (11).

Previous publications have demonstrated clinical improvement in lameness of horses treated with ACS or APS (9, 12), however, there is still little information of how they affect the cellular response in OA joints compared to corticosteroids. Synovial and cartilage explants cultured together have physiologic responses that closely resemble OA tissues *in situ* (13, 14). Comparing the effect of TA to orthobiologic products (ACS and APS) using a co-culture model may provide a better understanding of their effect in clinical cases.

The study objectives were: (1) To compare the cellular composition and concentration of important cytokines and growth factors within ACS and APS from the same individual horse, and (2) to investigate the effects of ACS, APS, and TA on inflammatory and catabolic gene expression in an IL-1β stimulated cartilage and synovial membrane co-culture model of OA. Our hypotheses were: (1) ACS and APS obtained from the same horses will have a different cellular and cytokine profile, (2) IL-1β would produce an inflammatory response in the co-cultured articular cartilage and synovial tissue, (3) TA would reduce expression and production of inflammatory proteins more effectively than orthobiologics (ACS and APS), but orthobiologics would protect matrix gene expression more effectively than TA. Gaining a better understanding of how these therapies work may help veterinarians make informed decisions on the use of these products to treat joint disease in horses.

## Materials and Methods

### Subjects

This study was performed in accordance with Institutional and NIH guidelines for the Care and Use of Laboratory Animals, and the study was approved by the Institutional Animal Care and Use Committee (IACUC) at Auburn University. Six adult American Quarter horses (1 mare and 5 geldings, aged 14.6 ± 4.99 years) free of systemic disease and euthanized for reasons unrelated to the study were used. Horses with history of lameness related to the stifle and/or stifle effusion were excluded from the study. Horses were deemed systemically healthy by physical examination and complete blood count.

### Orthobiologic Products Preparation

Blood was collected aseptically and processed according to the manufacturer's instructions to produce ACS (Orthokine® Overland Park, KS), and APS (Pro-Stride® Owl Manor, Warsaw IN).

For ACS, 60 mL of blood was aseptically collected from the jugular vein 24 h prior to euthanasia into an ACS syringe containing CrSO_4_-treated glass beads from the jugular vein. Blood was incubated at 37°C for 24 h then centrifuged at 3,000 RCF for 10 min and serum collected. A 3 mL aliquot of ACS was kept at 4°C after processing until use with culture media.

For APS, 104 mL of blood was aseptically collected into two syringes (52 mL of blood in each syringe) containing acid citrate dextrose (ACD-A) (Citra Labs, Baintree, MA) (8 mL in each syringe). Following collection, the blood was transferred to the APS separator and centrifuged (Owl Manor centrifuge, Owl Manor, Warsaw IN) at 3,200 RPM for 15 min. Platelet-poor plasma was removed, and the platelet–rich cell solution was transferred to the APS concentrator containing polyacrylamide beads and centrifuged at 2,000 RPM for 2 min. The volume obtained from one of the kits (3 mL) was kept at 4°C until use with culture media.

The remaining product for both ACS and APS was aliquoted, snap-frozen, and stored at −80°C for further analysis.

### Cellular, Cytokine, and Growth Factor Analysis of ACS and APS

The concentration of white blood cells (WBCs), red blood cells (RBCs), and platelet (PLT) counts were measured in blood as well as ACS and APS by hematologic analyzer (ADVIA® 120 Hematology System, Siemens). ELISA analysis was performed using commercially available kits (R&D Systems, Minneapolis, MN), previously validated in horses, for growth factor (TGF-β), anti-inflammatory (IL-1rap, and sTNF-R1), and pro-inflammatory (IL-1β, TNF-α, and MMP-3) cytokines (12, 15–17). Standards provided for the ELISA were used to prepare a standard curve following manufacturer's instructions. Samples were not diluted to measure IL-β and IL-1rap, while they were diluted at 1:40 to measure TGF-β, 1:4 to measure TNF-α and sTNF-R, and 1:10 to measure MMP-3. Cytokine measurements were performed in triplicate.

### Synovial Membrane and Tissue Harvesting

Following euthanasia, synovial membrane, and articular cartilage were aseptically harvested from the femoropatellar and femorotibial joints. Tissues from horses with gross signs of osteoarthritis including cartilage erosion, score lines, discoloration, or fibrillation (18) were not used. Also, the synovial membrane was evaluated for gross signs of synovitis such as hyperemia, hypertrophy or fibrosis, and horses showing any of these changes were not included in the study. A 4 mm diameter disposable biopsy punch (Integra, Saint Priest, France) was used to obtain the cartilage and synovial membrane explants. Twenty-eight cartilage explants were obtained from the medial and lateral femoral condyles of each horse. The synovial membrane was dissected from the fibrous joint capsule, and 36 explants were obtained.

### Co-culture

A co-culture was created by adding a hanging insert (WVR, Radnor, PA) containing 2 cartilage explants overtop of 3 synovial membrane explants in a 12-well culture plate. This ratio was calculated based on the ratios described for humans and mice, where synovial tissue has been shown to be 1.3x more plentiful than the articular cartilage surface in the synovial environment (19). For each treatment group, co-cultures were plated in duplicate. Cultures were maintained for 2 h under standard culture conditions with Dulbecco's Modified Eagle Medium (high glucose, 4,500 mg/L) with L-glutamine and sodium bicarbonate, free of sodium pyruvate (BioWhittaker; Lonza, Basel, Switzerland), supplemented with streptomycin (100 μg/ml) and penicillin (100 μg/mL) with 10% equine serum to allow tissue acclimatization. This short acclimatization was chosen so that the orthobiologics were not subject to cryopreservation prior to treatment. Incubation was maintained at 37°C and 5% CO_2_ room air incubator, in culture media as defined above. After 2 h, culture media was removed, tissues were rinsed with phosphate-buffered saline, and replaced in co-culture. Media was added to the culture according to the following conditions: (1) unstimulated control, (2) stimulated control, (3) ACS at 25% v/v (4) ACS at 50% v/v, (5) APS at 25% v/v, (6) APS at 50% v/v, (7) TA (10^−6^ M) (20). Groups 2–7 were stimulated with interleukin-1β (10 ng/ml) (R&D Systems, Minneapolis, MN). Cultures were maintained at 37°C with 5% CO_2_ for 96 h. At study termination, media was snap-frozen and stored at −80°C for later analysis. Cartilage and synovial explants were removed from the culture, rinsed in phosphate-buffered saline, snap-frozen in liquid nitrogen, and stored at −80°C.

### PGE_2_ Concentrations in Culture Media

A commercially available ELISA assay for PGE_2_ (R&D Systems, Minneapolis, MN) was used to measure PGE_2_ concentration in culture media according to manufacturer's instructions. This colorimetric assay was not equine-specific; however, it has been previously referenced and validated for cross-reactivity in equine samples (12, 21, 22). Standards provided for the ELISA were used to prepare a standard curve following manufacturer's instructions. Media samples were diluted at 1:30, and PGE_2_ measurements performed in triplicate.

### Gene Expression in Cartilage and Synovial Membrane

Frozen cartilage samples were added to TRIzol reagent (Invitrogen, Carlsbad, CA) then pulverized with a tissue homogenizer (Polytron, Thomas Scientific, Swedesboro, NJ). Synovial membrane samples were homogenized in TRIzol using a bead homogenizer (TissueLysser LT, Qiagen, Germantown, MD) for 20 min. To isolate RNA, a chloroform extraction protocol was performed. Briefly, 200 μl of chloroform were added to the samples, mixed vigorously, and incubated at room temperature for 10 min. The mix was centrifuged at 17,000 RCF for 15 min at 4°C. The supernatant was saved (approximately 500 μl), while the remaining pellet was discarded. Equal parts of isopropanol were added and incubated at −20°C for 15 min followed by a centrifugation at 17,000 RCF for 20 min. The supernatant was decanted, and 1 mL of 75% cold ethanol was added and spun at same speed for 10 min. The supernatant was decanted again, and once the ethanol was completely evaporated; the pellet was re-suspended on 40 μl of nuclease free water. Nucleic acid concentrations were determined using a spectrophotometer at 260/280 nm (DeNovix, Wilmington, DE). RNA was stored at −80°C until qPCR analysis. RNA was reverse transcribed to cDNA using iScript™ gDNA Clear cDNA Synthesis Kit (Bio-Rad, Hercules, California). Relative gene expression of IL-1β, MMP-1, MMP-3, MMP-13, IL-6, IL-8, IL-10, and ADAMTS-4 (in synovial membrane) and type II collagen (COL2A1), aggrecan (ACAN), TNF-α, IL-1β, and ADAMTS-4 (in articular cartilage) was calculated. All primers were derived from the Equus caballus genome (GenBank) and designed using the NCBI-Primer-BLAST (Table 1). Primer efficiencies were determined using 2-fold dilutions of cDNA and efficiencies calculated for all the primers ranged between 94 and 102.5%. All the qPCR experiments were performed in triplicate using SYBR Green Master Mix (PerfeCTa SYBR Green FastMix, Quantabio, Beverly, Massachusetts). The thermocycler (CFX 96 Thermocycler Bio-Rad Hercules, California) was heated at 95°C for 15 min, followed by 40 cycles of 94°C for 15 s, 55°C for 30 s, and 70°C for 30s, followed by a melting curve analysis. The relative gene expression was calculated by the comparative threshold cycle method (ΔΔCt method). Reference genes used were 18s and SCAMP3 for synovial membrane and GAPDH and SCAMP3 for articular cartilage. These genes were selected by evaluating the stability of various reference genes with equine tissue (18-S, β2M, GAPDH, SDHA, HPRT1, SCAMP-3, and β-actin). ΔΔCt values for all these genes were calculated under different stimulatory conditions in the 6 horses and the two genes for each tissue with the least amount of change in gene expression were chosen (23). Differences in gene expression were determined as fold change of relative gene expression of the control tissues compared to the IL-1β stimulation group and IL-1β stimulation group compared to treatment groups.

**Table 1:** Equine primer sequences used for gene expression analyses.

**Gene**		**Primer sequence**
18 small ribonucleic acid (18S)	Forward	5′- GCCGCTAGAGGTGAAATTCT-3′
	Reverse	5′- TCGGAACTACGACGGTATCT−3′
Secretory Carrier Membrane Protein 3 (SCAMP 3)	Forward	5′-CTGTGCTGGGAATTGTGATG-3′
	Reverse	5′-ATTCTTGCTGGGCCTTCTG-3′
Glyceraldehyde 3-phosphate dehydrogenase (GAPDH)	Forward	5′-GTCATCAACGGAAAGGC-3′
	Reverse	5′-GCATCAGCAGAAGGAGCA-3′
Interleukin-1 β (IL-1β)	Forward	5′-GCGGCAATGAGAATGaCCTG-3′
	Reverse	5′-AGCCACAATGATTGACACGA-3′
Interleukin-6 (IL-6)	Forward	5′-AACAGCAAGGAGGTACTGGCA-3′
	Reverse	5′-CAGGTCTCCTGATTGAACCCA-3′
Interleukin-8 (IL-8)	Forward	5′-AGGGACAGCAGAGACAGAGACACAAG-3′
	Reverse	5′-TACAACCGCAGCTTCACACA-3′
Interleukin-10 (IL-10)	Forward	5′-GCCTTGTCGGAGATGATCCA-3′
	Reverse	5′-TTTTCCCCCAGGGAGTTCAC-3′
Matrix MMETALLOPROTEINASE (MMP-1)	Forward	5′-GGTGAAGGAAGGTCAAGTTCTGAT-3′
	Reverse	5′-AGTCTTCTACTTTGGAAAAGAGCTTCTC-3′
Matrix metalloproteinase 3 (MMP- 3)	Forward	5′-GGCAACGTAGAGCTGAGTAAAGCC-3′
	Reverse	5′-CAACGGATAGGCTGAGCACGC-3′
Matrix metalloproteinase 13 (MMP-13)	Forward	5′-GTCCCTGATGTGGGTGAATAC-3′
	Reverse	5′-ACATCAGACAAACTTTGAAGG-3′
Tumor necrosis factor (TNF-α)	Forward	5′-AAAGGACATCATGAGCACTGAAAG-3′
	Reverse	5′-GGGCCCCCTGCCTCCT-3′
ADAM metallopeptidase with thrombospondin type 1 motif 4 (ADAMTS-4)	Forward	5′-GCTGTGCTATTGTGGAGGATGATGG-3′
	Reverse	5′-CCAGGGAAAGTCACAGGCAGATG-3′
Aggrecan (ACAN)	Forward	5′-CCTTGACTCCAGTGGTCTTATC-3′
	Reverse	5′GTCGTGGACCACCTAATTCTATC-3′
Type II collagen (COL2)	Forward	5′-GCCCGTCTGCTTCTTGTAATA-3′
	Reverse	5-CGTGACTGGGATTGGAAAGT-3′

### Statistical Analysis

All gene expression data were naturally log-transformed prior to analysis to equalize variances. Linear mixed models were used to analyze cytokine, growth factor, and cellular concentrations as well as gene expression. The full model for each concentration or gene expression variable included a fixed factor for condition and a random estimate for each horse. The random intercept for each horse accounted for within horse correlation. Model residuals were examined to evaluate the assumption of normality. Multiple comparisons were adjusted and analyzed using Tukey's test. Satterthwaite degrees of freedom method and restricted maximum likelihood (REML) estimation were used to evaluate significance. A Pearson correlation test was performed to evaluate the correlation between the cellular composition and cellular proteins. All analyses were performed using SAS V 9.4 (Cary, NC). Significance was set at *p* < 0.05.

## Results

### Cellular, Cytokine, and Growth Factor Analysis of ACS and APS

The cellular composition of peripheral blood, ACS and APS varied significantly between products (Table 2). ACS had significantly lower RBC (*p* = 0.001), and lower, but no significantly lower WBC and PLT counts compared to blood (*p* = 0.2803 and *p* = 0.140). APS had significantly greater WBCs (*p* < 0.001) and PLTs (*p* = 0.001), but lower RBCs (*p* < 0.001) compared to peripheral blood. APS had significantly greater WBCs (*p* < 0.001), RBCs (*p* = 0.0214) and PLTs (*p* < 0.001) compared to ACS.

**Table 2:** Summary of the cellular components of ACS and APS.

**Cellular component**	**Blood**	**ACS**	**ACS:Blood ratio**	**APS**	**APS:Blood ratio**
WBC count (x10^6^/ml)	7.57 ± 1.01	0.06 ± 0.04	0.008	35.37 ± 14.16^*^^#^	4.9
Platelet count (x10^6^/ml)	182.67 ± 56.53	4.5 ± 1.87	0.02	647.5 ± 257.65^*^^#^	3.5
RBC count (x10^6^/ml)	8.44 ± 0.83	0.01 ± 0.01^*^	0.001	0.9 ± 0.29^*^^#^	0.1

* *Significant p < 0.005 difference from blood values*.

# *Significant P < 0.005 difference from ACS*.

**Figure 1:**
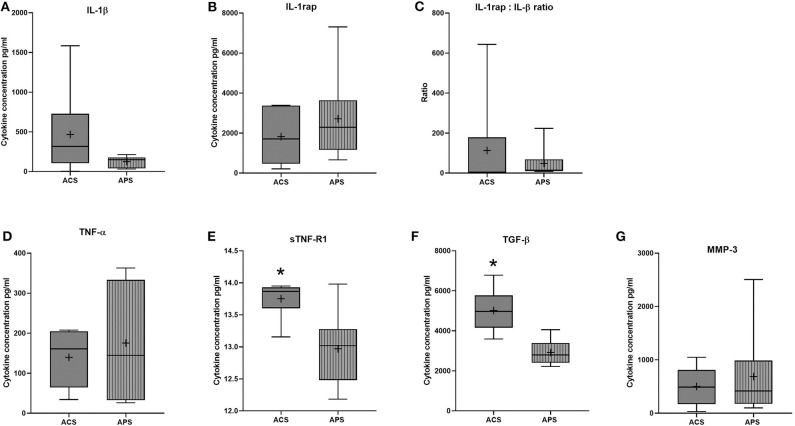
ACS and APS concentrations of **(A)** IL-1β (pg/ml), **(B)** IL-1rap (pg/ml), **(C)** IL-1rap: IL-1β ratio, **(D)** TNF-α (pg/ml), **(E)** sTNF-R1 (pg/ml), **(F)** TGF-β (pg/ml), and **(G)** MMP-3 (pg/ml). The boxplots represent the interquartile range (IQR) of n = 6. The black lines represent the median values, the crosses represent the mean, and the whiskers represent the values outside the IQR. *Denotes significant difference between ACS and APS, *p* < 0.05.

Few differences in cytokines and growth factors were observed between ACS and APS (Figure 1). Variability between horses in cytokines and growth factor concentrations was observed, but this variability between horses was only significantly different for TNFα (*p* = 0.001). No significant differences in concentrations of IL-1β, TNFα, MMP-3, and IL-1rap between products were observed. However, TGF-β (*p* = 0.009) and sTNF-R1 (*p* = 0.010) were significantly increased in ACS compared to APS. When the ratio of IL-1rap: IL-1β ratio was evaluated for each individual horse, ACS (113.31 ± 78.98) had a higher ratio compared to APS (48.22 ± 78.98), but this difference was not significant (*p* = 0.401). Positive and negative correlations between cytokines and cellular components were found (Table 3).

**Table 3:** Summary of the significant correlations between cytokines, growth factor and cellular components.

**Cellular, cytokine, and growth factor components correlation**	**Pearson's correlation coefficient**	***P*- value**
IL-1β and MMP-3	0.5088	*p* = 0.0311
IL-1β and TGF-β	0.7018	*p* = 0.0110
WBC and TGF-β	−0.6186	*p* = 0.0320
PLTs and TGFβ	−0.6861	*p* = 0.0138
PLTs and sTNF-1R	−0.6573	*p* = 0.0202

### PGE_2_ Concentrations in Co-culture Media

**Figure 2:**
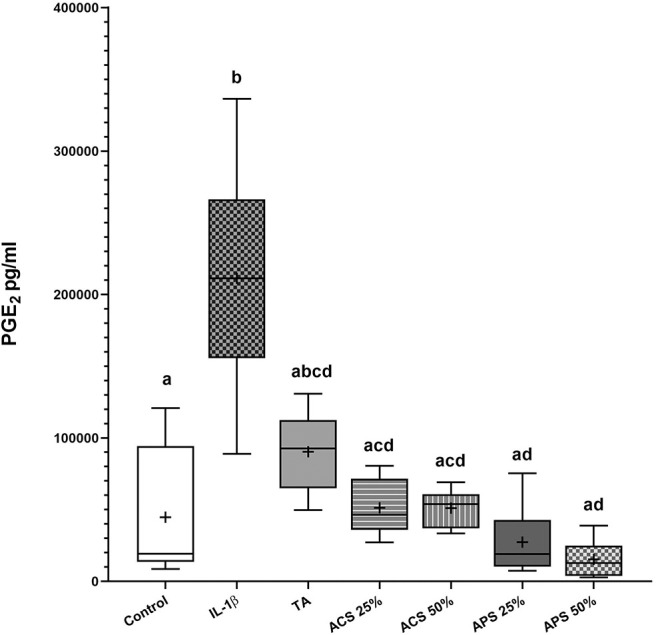
PGE2 concentrations in the co-culture media 96 hours after treatment. The boxplots represent the interquartile range (IQR) of n = 6. The black lines represent the median values, the crosses represent the mean, and the whiskers represent the values outside the IQR. Different letters denote significant differences between groups, *p* < 0.05.

All treatments reduced PGE_2_ concentrations in the co-culture media compared to stimulated controls (Figure 2). PGE_2_ concentration increased 4.7-fold (*p* = 0.028) after stimulation with IL-1β. PGE_2_ concentration was reduced but not significantly changed after TA treatment (*p* = 0.111), while media with ACS at 25% v/v and 50% v/v, decreased PGE_2_ concentration by 4.13-fold (*p* = 0.037 and *p* = 0.038 respectively). APS caused a dose dependent reduction in PGE_2_ concentrations following IL-1β stimulation, with a 7.7-fold reduction in PGE_2_ at 25% v/v (*p* = 0.019), and a 13.8-fold reduction in PGE_2_ at 50% v/v (*p* = 0.016). In summary, APS 50% v/v was the most effective, while TA was the least effective at reducing PGE_2_.

### Gene Expression

**Figure 3:**
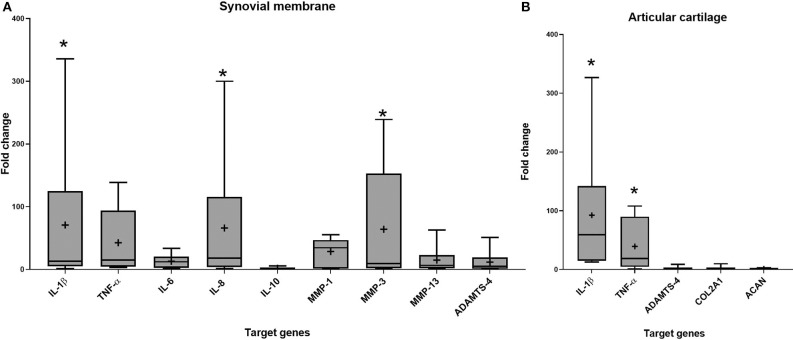
Fold change in relative gene expression comparing unstimulated tissues versus tissues stimulated with IL-1β in **(A)** synovial membrane tissue **(B)** articular cartilage tissue. The boxplots represent the interquartile range (IQR) of n = 6. The black lines represent the median values, the crosses represent the mean, and the whiskers represent the values outside the IQR. *Denotes significant difference between control group and stimulated IL-1β group gene expression, *p* < 0.05.

Gene expression was assessed in synovial membrane and articular cartilage after 96 h of co-culture. Stimulation with IL-1β upregulated IL-1β (*p* = 0.029), IL-8 (*p* = 0.011), and MMP-3 (*p* = 0.043) in synovial membrane and IL-1β (*p* = 0.003) and TNF-α (*p* = 0.005) in articular cartilage compared to the control groups (Figure 3).

**Figure 4:**
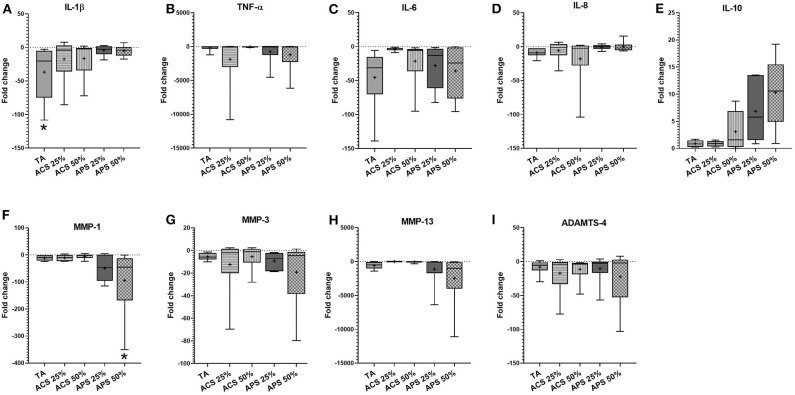
Fold change in relative gene expression comparing IL-1β stimulated synovial membrane with IL-1β stimulated tissue treated with triamcinolone (TA), autologous conditioned serum (ACS) and autologous protein solution (APS) of **(A)** TNF-α, **(B)** IL-1β, **(C)** IL-8, **(D)** IL-6, **(E)** IL-10, **(F)** MMP-1, **(G)** MMP-3, **(H)** MMP-13, and **(I)** ADAMTS-4. The boxplots represent the interquartile range (IQR) of n = 6. The black lines represent the median values, the crosses represent the mean, and the whiskers represent the values outside the IQR. *Denotes significant difference between stimulation IL-1β group and treatment, *p* < 0.05.

In synovial membrane, TA was the only treatment that reduced IL-1β expression significantly (*p* = 0.011) and trended to reduce the expression of IL-6 (*p* = 0.402) more effectively than ACS (*p* = 0.780) or APS (*p* = 0.601). APS at 50% v/v significantly reduced expression of the matrix degrading enzyme MMP-1 (*p* = 0.025), and showed a trend to reduce MMP-3 (*p* = 0.275), MMP-13 (*p* = 0.140), and ADAMTS-4 (*p* = 0.158) expression. Treatment with ACS and APS showed a trend to upregulate IL-10 gene expression more effectively than TA (*p* = 0.670 and *p* = 0.452 respectively), but did not downregulate IL-6 as effectively as TA (*p* = 0.402) (Figure 4).

**Figure 5:**
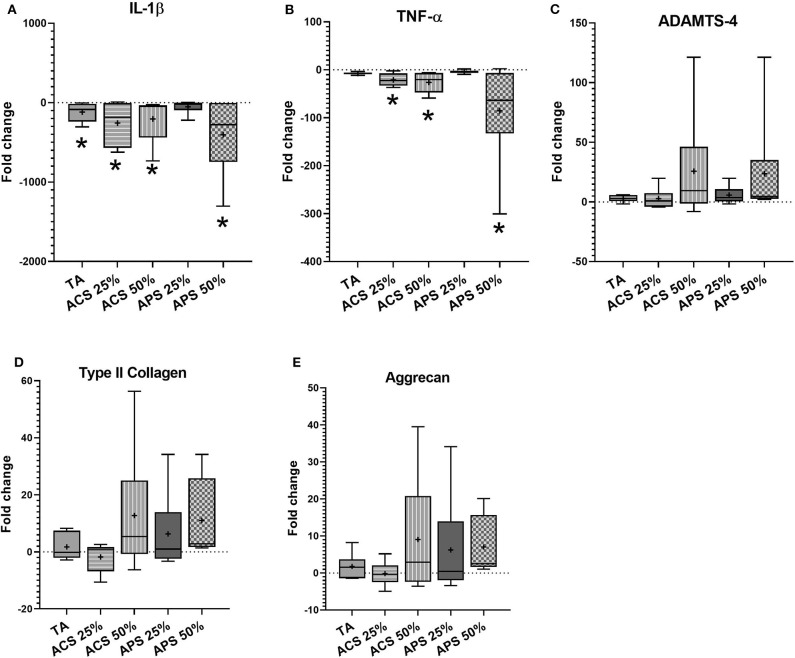
Fold change in relative gene expression comparing IL-1β stimulated articular cartilage with IL-1β stimulated tissue treated with triamcinolone (TA), autologous conditioned serum (ACS) and autologous protein solution (APS) of **(A)** TNF-α, **(B)** IL-1β, **(C)** ADAMTS-4, **(D)** COL2A1, and **(E)** Aggrecan. The boxplots represent the interquartile range (IQR) of n = 6. The black lines represent the median values, the cross represents the mean, and the whiskers represent the values outside the IQR. *Denotes significant difference between stimulation IL-1β group and treatment, *p* < 0.05.

In articular cartilage, ACS and APS treatments caused significant downregulation of IL-1β expression (*p* = 0.001), with 10-fold greater downregulation than TA (*p* = 0.002) compared to the stimulated control group. In addition, treatment with 50% v/v ACS and APS downregulated TNF-α gene expression (*p* = 0.014 and *p* = 0.002, respectively). ACS and APS showed a trend toward upregulation of ACAN (*p* = 0.549 and *p* = 0.529, respectively) and COL2A1 (*p* = 0.678 and *p* = 0.526, respectively). Treatment with ACS and APS trended toward upregulation of ADAMTS-4 expression (*p* = 0.309 and *p* = 0.315, respectively). A dose-effect was observed with ACS and APS treatment, with 50% v/v treatment producing greater effect on the expression of TNF-α, ADAMTS-4, COL2A1 and ACAN vs. 25% v/v (Figure 5).

## Discussion

This is the first study comparing corticosteroids to orthobiologic therapies in an equine *in vitro* co-culture model of OA. As we hypothesized, TA was more efficient at downregulating IL-1β expression in the synovial membrane. Although not significant, ACS and APS produced an upregulation of important matrix proteins, COL2A1 and ACAN, and downregulation of inflammatory genes, IL-1β and TNF-α in articular cartilage, changes that might offer protection of the articular cartilage. ACS and APS also modified the inflammatory response by increasing the gene expression of the anti-inflammatory cytokine IL-10 and decreasing the concentration of PGE_2._ Additionally, APS downregulated the expression of MMP-1 in synovial membrane, which is one of the main collagenases produced primarily by the synovial cells (24).

Orthobiologic treatments, ACS and APS, aim to modify the inflammatory cascade to reduce cartilage destruction and improve endogenous repair mechanisms in OA. ACS has been shown to have disease-modifying properties in human and equine studies producing an improvement in clinical signs and modification of the cellular response (12, 25–27). Recently, an *in vivo* study found that treatment of ACS produced a disease-modifying effect decreasing cartilage biomarkers in horses with advanced OA (28). Clinically, a single injection of APS improved pain scores up to 1 year and reduced osteophyte formation in people (29–31). In horses and dogs, APS has improved pain scores and reduced lameness up to 1 year after treatment (9, 32). *In vitro*, limited anti-inflammatory effects have been observed following treatment of articular cells and/or tissues with APS (11, 33, 34).

ACS and APS differ in their processing methods, targeting different blood components for the concentration of cells, platelets, and proteins. The current study identified significant differences in cellular composition between ACS and APS, but few significant differences in measured cytokines and growth factors were identified. APS had higher WBCs compared to ACS and blood. The WBC, RBC, and PLT concentration of APS compared to blood has been previously reported (9) in the horse. The results of our study showed a smaller increase in WBCs (12.1 vs. 4.9-fold increase) and a greater increase in PLTs (1.6 vs. 3.5-fold increase) compared to what has been reported by Bertone et al., most likely due to individual variations in physiologic status. In our study, ACS produced a significantly higher concentration of sTNF-R1 (*p* = 0.009) and TGF-β (*p* = 0.024), compared to APS, while another publication found that APS produced a higher concentration of TGF-β compared to ACS (11). Significant differences in cytokine concentration using different commercial kits under different physiologic conditions from the same horse has been reported with ACS (8, 10), which could explain differences between studies.

Co-culture of IL-1β stimulated cartilage and synovium has been shown to produce tissue related changes that resemble changes in tissues from joints with natural, ongoing OA compared to monoculture of synovial cells and/or tissues (13, 14). The effects of ACS and APS have been previously studied in IL-1β stimulated chondrocytes, where APS resulted in an increased concentration of chondroprotective cytokines (IL-1rap and IL-10) compared to ACS treatment. However, in that study, orthobiologics were not compared to the standard articular treatment, corticosteroids (11).

Stimulation with IL-1β produced an inflammatory response, upregulating expression of IL-1β, TNF-α, IL-8 and MMP-3 in synovial membrane and IL-1β and TNF-α in articular cartilage as shown in other studies (11, 13, 27, 35). In our study, TA significantly downregulated IL-1β expression in synovial membrane and articular cartilage, as well as a trend to downregulate IL-6 in synovial membrane but did not reduce expression of other inflammatory genes such as TNFα and IL-8. ACS and APS downregulated the expression of IL-1β and TNF-α in articular cartilage and showed a trend to upregulate COL2A1 and ACAN more effectively than TA. Additionally, APS produced a downregulation of MMP-1 and a trend for downregulation of matrix degrading enzymes in synovial membrane. Elevated MMP-1 expression has been measured in horses with OA (36), and downregulation of this protein is essential to slow down the progress of OA disease (37). The effect on the matrix gene expression of ACS and APS has not been evaluated previously. However, other disease-modifying effects have been reported. APS inhibited IL-1α and TNFα stimulated matrix degradation of bovine articular cartilage explants compared to direct recombinant antagonists (IL-1rap and sTNF-R1) (34) and downregulated MMP-13 in human chondrocytes stimulated with IL-1β and TNF-α (33). In our study, both ACS and APS produced a trend to upregulate ADAMST-4 in articular cartilage, but not in synovial membrane. ADAMST-4 participates in aggrecan cleavage (38–40). However, other publications have found that ADAMTS-5 could have more significant effects on articular degradation (41, 42). Cartilage and meniscal explants cultured with double spin platelet-rich plasma (PRP) showed an upregulation of ADAMTS-4 compared to single spin PRP, suggesting that high platelet concentrations in PRP may produce a pro-inflammatory environment for cartilage (43). In our study, ACS and APS both produced a similar trend to upregulate ADAMTS-4 expression in cartilage and downregulate it in the synovial membrane, despite the differences in platelet concentration. It is possible that cross-talk between tissues is occurring and changes in expression of this protein does not happen within these two tissues at the same time. No studies have evaluated the effect of orthobiologics on ADAMST-4 and 5 expression in long-term synovial tissue culture, further investigation in this direction is warranted.

PGE_2_ is one of the primary pro-inflammatory mediators that promote catabolic destruction of articular cartilage as well as promotion of joint pain (44). Our study showed that APS reduced the production of PGE_2_ in IL-1β stimulated tissues by 13-fold compared to the stimulated control group, while TA only reduced PGE_2_ by 2.3-fold compared to the stimulated control group. *In vitro*, IL-1β stimulates PGE_2_ production (13, 45), decreasing the expression of IL-1β could lead to decreased concentration of IL-1β induced PGE_2_ production. Additionally, IL-10 is considered to be an essential anti-inflammatory cytokine participating in the downregulation of PGE_2_ (46– 48)_._ Linardi et al. evaluated the effects of APS treatment on equine chondrocytes demonstrating enhanced IL-10, IL-1rap, and IL-6 production compared to ACS in a standard chondrocyte culture (11). An association between a low PGE_2_ concentration in culture media, with upregulation of IL-10 gene expression, was observed in ACS and APS. This correlation leads us to hypothesize that IL-10 upregulation produced by ACS and APS will produce a decreased production of PGE_2._ In humans, PGE_2_ has been shown to sensitize nociceptor neurons (49). Therefore, downregulation of this protein may explain the clinical improvement in observed lameness in horses treated with ACS or APS (9, 12, 28, 50).

This study was limited by its *ex vivo* model as well as the inherent biologic variation in tissues and their response between horses. Variability between horses in inflammatory and anti-inflammatory mediators (cytokines and growth factors) was identified in ACS and APS, which led to unstandardized treatment between horses. However, this study was designed to reflect the clinical situation in which a horse's blood would be processed and used to treat their own joint, leading to a variable clinical response based on the composition of the biologic and degree of tissue pathology. The short-term model could be a disadvantage to fully understand how the cellular response to orthobiologic products changes with time. In humans, better clinical outcomes following treatment with APS have been observed 6-months post-treatment compared to 3 months post-treatment in patients with knee OA (31). Therefore, a short-term model may not fully explain the extent of modification that occurs with these products on the cellular and tissue response over time. This study primarily focuses on modification of gene expression produced. Ideally, changes in protein expression would be correlated with protein concentration in the media to validate the importance of changes in gene expression observed. In our study, IL-1β was the only cytokine used to produce an inflammatory response, while other *in vitro* studies have used a combination of IL-1β and TNF-α (11, 14). This could produce a different inflammatory response and account for differences in observed results.

In summary, TA downregulated the expression of IL-1β in synovial membrane, however, ACS and APS produced a stronger anti-inflammatory effect, modulating pro-inflammatory cytokines (IL-1β and TNF-α) involved in cartilage destruction in OA (51). ACS and APS, showed a chondroprotective effect by upregulating matrix gene expression, while TA treatment did not modify gene expression. Both ACS and APS significantly decreased PGE_2_ in media compared to TA, which could be one of the reasons horses with naturally occurring OA show improvement in lameness after treatment with ACS or APS. Since cartilage is characterized by its poor intrinsic capacity for repair, treatments that slow down the degenerative response and increase the reparative response would be ideal in treatment of OA. Considering the results of our study, the significant PGE_2_ reduction in media and the downregulation of pro-inflammatory cytokines, ACS and APS may provide important benefits in early stages of OA, slowing down the catabolic process occurring within the joint.

## Data Availability Statement

The raw data supporting the conclusions of this article will be made available by the authors, without undue reservation, to any qualified researcher.

## Ethics Statement

The animal study was reviewed and approved by Institutional Animal Care and Use Committee (IACUC) at Auburn University.

## Author Contributions

LB and AV conceived and planned the experiments. AV carried out the experiments. AW, LB, SP, FC, and AV contributed to sample preparation. AW, SP, LB, and AV contributed to the interpretation of the results. AV took the lead in writing the manuscript. All authors provided critical feedback and helped shape the research, analysis and manuscript.

## Conflict of Interest

The authors declare that the research was conducted in the absence of any commercial or financial relationships that could be construed as a potential conflict of interest.
